# 1918 Influenza: the Mother of All Pandemics

**DOI:** 10.3201/eid1201.050979

**Published:** 2006-01

**Authors:** Jeffery K. Taubenberger, David M. Morens

**Affiliations:** * Armed Forces Institute of Pathology, Rockville, Maryland, USA;; † National Institutes of Health, Bethesda, Maryland, USA

**Keywords:** influenza, pathogenesis, history, pandemic

## Abstract

The "Spanish" influenza pandemic of 1918–1919, which caused ≈50 million deaths worldwide, remains an ominous warning to public health. Many questions about its origins, its unusual epidemiologic features, and the basis of its pathogenicity remain unanswered. The public health implications of the pandemic therefore remain in doubt even as we now grapple with the feared emergence of a pandemic caused by H5N1 or other virus. However, new information about the 1918 virus is emerging, for example, sequencing of the entire genome from archival autopsy tissues. But, the viral genome alone is unlikely to provide answers to some critical questions. Understanding the 1918 pandemic and its implications for future pandemics requires careful experimentation and in-depth historical analysis.

"Curiouser and curiouser!" cried Alice

Lewis Carroll, Alice's Adventures in Wonderland, 1865

An estimated one third of the world's population (or ≈500 million persons) were infected and had clinically apparent illnesses (*1**,**2*) during the 1918–1919 influenza pandemic. The disease was exceptionally severe. Case-fatality rates were >2.5%, compared to <0.1% in other influenza pandemics (*3**,**4*). Total deaths were estimated at ≈50 million (*5**–**7*) and were arguably as high as 100 million (*7*).

The impact of this pandemic was not limited to 1918–1919. All influenza A pandemics since that time, and indeed almost all cases of influenza A worldwide (excepting human infections from avian viruses such as H5N1 and H7N7), have been caused by descendants of the 1918 virus, including "drifted" H1N1 viruses and reassorted H2N2 and H3N2 viruses. The latter are composed of key genes from the 1918 virus, updated by subsequently incorporated avian influenza genes that code for novel surface proteins, making the 1918 virus indeed the "mother" of all pandemics.

In 1918, the cause of human influenza and its links to avian and swine influenza were unknown. Despite clinical and epidemiologic similarities to influenza pandemics of 1889, 1847, and even earlier, many questioned whether such an explosively fatal disease could be influenza at all. That question did not begin to be resolved until the 1930s, when closely related influenza viruses (now known to be H1N1 viruses) were isolated, first from pigs and shortly thereafter from humans. Seroepidemiologic studies soon linked both of these viruses to the 1918 pandemic (*8*). Subsequent research indicates that descendants of the 1918 virus still persists enzootically in pigs. They probably also circulated continuously in humans, undergoing gradual antigenic drift and causing annual epidemics, until the 1950s. With the appearance of a new H2N2 pandemic strain in 1957 ("Asian flu"), the direct H1N1 viral descendants of the 1918 pandemic strain disappeared from human circulation entirely, although the related lineage persisted enzootically in pigs. But in 1977, human H1N1 viruses suddenly "reemerged" from a laboratory freezer (*9*). They continue to circulate endemically and epidemically.

Thus in 2006, 2 major descendant lineages of the 1918 H1N1 virus, as well as 2 additional reassortant lineages, persist naturally: a human epidemic/endemic H1N1 lineage, a porcine enzootic H1N1 lineage (so-called classic swine flu), and the reassorted human H3N2 virus lineage, which like the human H1N1 virus, has led to a porcine H3N2 lineage. None of these viral descendants, however, approaches the pathogenicity of the 1918 parent virus. Apparently, the porcine H1N1 and H3N2 lineages uncommonly infect humans, and the human H1N1 and H3N2 lineages have both been associated with substantially lower rates of illness and death than the virus of 1918. In fact, current H1N1 death rates are even lower than those for H3N2 lineage strains (prevalent from 1968 until the present). H1N1 viruses descended from the 1918 strain, as well as H3N2 viruses, have now been cocirculating worldwide for 29 years and show little evidence of imminent extinction.

## Trying To Understand What Happened

By the early 1990s, 75 years of research had failed to answer a most basic question about the 1918 pandemic: why was it so fatal? No virus from 1918 had been isolated, but all of its apparent descendants caused substantially milder human disease. Moreover, examination of mortality data from the 1920s suggests that within a few years after 1918, influenza epidemics had settled into a pattern of annual epidemicity associated with strain drifting and substantially lowered death rates. Did some critical viral genetic event produce a 1918 virus of remarkable pathogenicity and then other critical genetic event occur soon after the 1918 pandemic to produce an attenuated H1N1 virus?

In 1995, a scientific team identified archival influenza autopsy materials collected in the autumn of 1918 and began the slow process of sequencing small viral RNA fragments to determine the genomic structure of the causative influenza virus (*10*). These efforts have now determined the complete genomic sequence of 1 virus and partial sequences from 4 others. The primary data from the above studies (*11**–**17*) and a number of reviews covering different aspects of the 1918 pandemic have recently been published (*18**–**20*) and confirm that the 1918 virus is the likely ancestor of all 4 of the human and swine H1N1 and H3N2 lineages, as well as the "extinct" H2N2 lineage. No known mutations correlated with high pathogenicity in other human or animal influenza viruses have been found in the 1918 genome, but ongoing studies to map virulence factors are yielding interesting results. The 1918 sequence data, however, leave unanswered questions about the origin of the virus (*19*) and about the epidemiology of the pandemic.

## When and Where Did the 1918 Influenza Pandemic Arise?

Before and after 1918, most influenza pandemics developed in Asia and spread from there to the rest of the world. Confounding definite assignment of a geographic point of origin, the 1918 pandemic spread more or less simultaneously in 3 distinct waves during an ≈12-month period in 1918–1919, in Europe, Asia, and North America (the first wave was best described in the United States in March 1918). Historical and epidemiologic data are inadequate to identify the geographic origin of the virus (*21*), and recent phylogenetic analysis of the 1918 viral genome does not place the virus in any geographic context (*19*).

**Figure 1:**
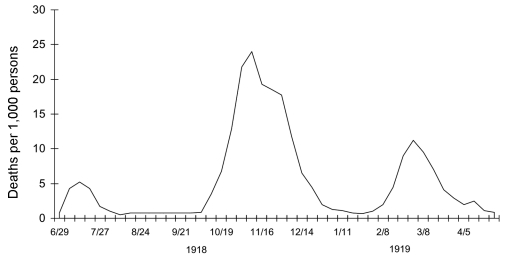
Three pandemic waves: weekly combined influenza and pneumonia mortality, United Kingdom, 1918–1919 (*21*).

Although in 1918 influenza was not a nationally reportable disease and diagnostic criteria for influenza and pneumonia were vague, death rates from influenza and pneumonia in the United States had risen sharply in 1915 and 1916 because of a major respiratory disease epidemic beginning in December 1915 (*22*). Death rates then dipped slightly in 1917. The first pandemic influenza wave appeared in the spring of 1918, followed in rapid succession by much more fatal second and third waves in the fall and winter of 1918–1919, respectively (Figure 1). Is it possible that a poorly-adapted H1N1 virus was already beginning to spread in 1915, causing some serious illnesses but not yet sufficiently fit to initiate a pandemic? Data consistent with this possibility were reported at the time from European military camps (*23*), but a counter argument is that if a strain with a new hemagglutinin (HA) was causing enough illness to affect the US national death rates from pneumonia and influenza, it should have caused a pandemic sooner, and when it eventually did, in 1918, many people should have been immune or at least partially immunoprotected. "Herald" events in 1915, 1916, and possibly even in early 1918, if they occurred, would be difficult to identify.

The 1918 influenza pandemic had another unique feature, the simultaneous (or nearly simultaneous) infection of humans and swine. The virus of the 1918 pandemic likely expressed an antigenically novel subtype to which most humans and swine were immunologically naive in 1918 (*12**,**20*). Recently published sequence and phylogenetic analyses suggest that the genes encoding the HA and neuraminidase (NA) surface proteins of the 1918 virus were derived from an avianlike influenza virus shortly before the start of the pandemic and that the precursor virus had not circulated widely in humans or swine in the few decades before (*12**,**15**,**24*). More recent analyses of the other gene segments of the virus also support this conclusion. Regression analyses of human and swine influenza sequences obtained from 1930 to the present place the initial circulation of the 1918 precursor virus in humans at approximately 1915–1918 (*20*). Thus, the precursor was probably not circulating widely in humans until shortly before 1918, nor did it appear to have jumped directly from any species of bird studied to date (*19*). In summary, its origin remains puzzling.

## Were the 3 Waves in 1918–1919 Caused by the Same Virus? If So, How and Why?

Historical records since the 16th century suggest that new influenza pandemics may appear at any time of year, not necessarily in the familiar annual winter patterns of interpandemic years, presumably because newly shifted influenza viruses behave differently when they find a universal or highly susceptible human population. Thereafter, confronted by the selection pressures of population immunity, these pandemic viruses begin to drift genetically and eventually settle into a pattern of annual epidemic recurrences caused by the drifted virus variants.

In the 1918–1919 pandemic, a first or spring wave began in March 1918 and spread unevenly through the United States, Europe, and possibly Asia over the next 6 months (Figure 1). Illness rates were high, but death rates in most locales were not appreciably above normal. A second or fall wave spread globally from September to November 1918 and was highly fatal. In many nations, a third wave occurred in early 1919 (*21*). Clinical similarities led contemporary observers to conclude initially that they were observing the same disease in the successive waves. The milder forms of illness in all 3 waves were identical and typical of influenza seen in the 1889 pandemic and in prior interpandemic years. In retrospect, even the rapid progressions from uncomplicated influenza infections to fatal pneumonia, a hallmark of the 1918–1919 fall and winter waves, had been noted in the relatively few severe spring wave cases. The differences between the waves thus seemed to be primarily in the much higher frequency of complicated, severe, and fatal cases in the last 2 waves.

But 3 extensive pandemic waves of influenza within 1 year, occurring in rapid succession, with only the briefest of quiescent intervals between them, was unprecedented. The occurrence, and to some extent the severity, of recurrent annual outbreaks, are driven by viral antigenic drift, with an antigenic variant virus emerging to become dominant approximately every 2 to 3 years. Without such drift, circulating human influenza viruses would presumably disappear once herd immunity had reached a critical threshold at which further virus spread was sufficiently limited. The timing and spacing of influenza epidemics in interpandemic years have been subjects of speculation for decades. Factors believed to be responsible include partial herd immunity limiting virus spread in all but the most favorable circumstances, which include lower environmental temperatures and human nasal temperatures (beneficial to thermolabile viruses such as influenza), optimal humidity, increased crowding indoors, and imperfect ventilation due to closed windows and suboptimal airflow.

However, such factors cannot explain the 3 pandemic waves of 1918–1919, which occurred in the spring-summer, summer-fall, and winter (of the Northern Hemisphere), respectively. The first 2 waves occurred at a time of year normally unfavorable to influenza virus spread. The second wave caused simultaneous outbreaks in the Northern and Southern Hemispheres from September to November. Furthermore, the interwave periods were so brief as to be almost undetectable in some locales. Reconciling epidemiologically the steep drop in cases in the first and second waves with the sharp rises in cases of the second and third waves is difficult. Assuming even transient postinfection immunity, how could susceptible persons be too few to sustain transmission at 1 point and yet enough to start a new explosive pandemic wave a few weeks later? Could the virus have mutated profoundly and almost simultaneously around the world, in the short periods between the successive waves? Acquiring viral drift sufficient to produce new influenza strains capable of escaping population immunity is believed to take years of global circulation, not weeks of local circulation. And having occurred, such mutated viruses normally take months to spread around the world.

At the beginning of other "off season" influenza pandemics, successive distinct waves within a year have not been reported. The 1889 pandemic, for example, began in the late spring of 1889 and took several months to spread throughout the world, peaking in northern Europe and the United States late in 1889 or early in 1890. The second recurrence peaked in late spring 1891 (more than a year after the first pandemic appearance) and the third in early 1892 (*21*). As was true for the 1918 pandemic, the second 1891 recurrence produced of the most deaths. The 3 recurrences in 1889–1892, however, were spread over >3 years, in contrast to 1918–1919, when the sequential waves seen in individual countries were typically compressed into ≈8–9 months.

What gave the 1918 virus the unprecedented ability to generate rapidly successive pandemic waves is unclear. Because the only 1918 pandemic virus samples we have yet identified are from second-wave patients (*16*), nothing can yet be said about whether the first (spring) wave, or for that matter, the third wave, represented circulation of the same virus or variants of it. Data from 1918 suggest that persons infected in the second wave may have been protected from influenza in the third wave. But the few data bearing on protection during the second and third waves after infection in the first wave are inconclusive and do little to resolve the question of whether the first wave was caused by the same virus or whether major genetic evolutionary events were occurring even as the pandemic exploded and progressed. Only influenza RNA–positive human samples from before 1918, and from all 3 waves, can answer this question.

## What Was the Animal Host Origin of the Pandemic Virus?

Viral sequence data now suggest that the entire 1918 virus was novel to humans in, or shortly before, 1918, and that it thus was not a reassortant virus produced from old existing strains that acquired 1 or more new genes, such as those causing the 1957 and 1968 pandemics. On the contrary, the 1918 virus appears to be an avianlike influenza virus derived in toto from an unknown source (*17**,**19*), as its 8 genome segments are substantially different from contemporary avian influenza genes. Influenza virus gene sequences from a number of fixed specimens of wild birds collected circa 1918 show little difference from avian viruses isolated today, indicating that avian viruses likely undergo little antigenic change in their natural hosts even over long periods (*24**,**25*).

For example, the 1918 nucleoprotein (NP) gene sequence is similar to that of viruses found in wild birds at the amino acid level but very divergent at the nucleotide level, which suggests considerable evolutionary distance between the sources of the 1918 NP and of currently sequenced NP genes in wild bird strains (*13**,**19*). One way of looking at the evolutionary distance of genes is to compare ratios of synonymous to nonsynonymous nucleotide substitutions. A synonymous substitution represents a silent change, a nucleotide change in a codon that does not result in an amino acid replacement. A nonsynonymous substitution is a nucleotide change in a codon that results in an amino acid replacement. Generally, a viral gene subjected to immunologic drift pressure or adapting to a new host exhibits a greater percentage of nonsynonymous mutations, while a virus under little selective pressure accumulates mainly synonymous changes. Since little or no selection pressure is exerted on synonymous changes, they are thought to reflect evolutionary distance.

Because the 1918 gene segments have more synonymous changes from known sequences of wild bird strains than expected, they are unlikely to have emerged directly from an avian influenza virus similar to those that have been sequenced so far. This is especially apparent when one examines the differences at 4-fold degenerate codons, the subset of synonymous changes in which, at the third codon position, any of the 4 possible nucleotides can be substituted without changing the resulting amino acid. At the same time, the 1918 sequences have too few amino acid differences from those of wild-bird strains to have spent many years adapting only in a human or swine intermediate host. One possible explanation is that these unusual gene segments were acquired from a reservoir of influenza virus that has not yet been identified or sampled. All of these findings beg the question: where did the 1918 virus come from?

In contrast to the genetic makeup of the 1918 pandemic virus, the novel gene segments of the reassorted 1957 and 1968 pandemic viruses all originated in Eurasian avian viruses (*26*); both human viruses arose by the same mechanism—reassortment of a Eurasian wild waterfowl strain with the previously circulating human H1N1 strain. Proving the hypothesis that the virus responsible for the 1918 pandemic had a markedly different origin requires samples of human influenza strains circulating before 1918 and samples of influenza strains in the wild that more closely resemble the 1918 sequences.

## What Was the Biological Basis for 1918 Pandemic Virus Pathogenicity?

Sequence analysis alone does not offer clues to the pathogenicity of the 1918 virus. A series of experiments are under way to model virulence in vitro and in animal models by using viral constructs containing 1918 genes produced by reverse genetics.

Influenza virus infection requires binding of the HA protein to sialic acid receptors on host cell surface. The HA receptor-binding site configuration is different for those influenza viruses adapted to infect birds and those adapted to infect humans. Influenza virus strains adapted to birds preferentially bind sialic acid receptors with α (2–3) linked sugars (*27**–**29*). Human-adapted influenza viruses are thought to preferentially bind receptors with α (2–6) linkages. The switch from this avian receptor configuration requires of the virus only 1 amino acid change (*30*), and the HAs of all 5 sequenced 1918 viruses have this change, which suggests that it could be a critical step in human host adaptation. A second change that greatly augments virus binding to the human receptor may also occur, but only 3 of 5 1918 HA sequences have it (*16*).

This means that at least 2 H1N1 receptor-binding variants cocirculated in 1918: 1 with high-affinity binding to the human receptor and 1 with mixed-affinity binding to both avian and human receptors. No geographic or chronologic indication exists to suggest that one of these variants was the precursor of the other, nor are there consistent differences between the case histories or histopathologic features of the 5 patients infected with them. Whether the viruses were equally transmissible in 1918, whether they had identical patterns of replication in the respiratory tree, and whether one or both also circulated in the first and third pandemic waves, are unknown.

In a series of in vivo experiments, recombinant influenza viruses containing between 1 and 5 gene segments of the 1918 virus have been produced. Those constructs bearing the 1918 HA and NA are all highly pathogenic in mice (*31*). Furthermore, expression microarray analysis performed on whole lung tissue of mice infected with the 1918 HA/NA recombinant showed increased upregulation of genes involved in apoptosis, tissue injury, and oxidative damage (*32*). These findings are unexpected because the viruses with the 1918 genes had not been adapted to mice; control experiments in which mice were infected with modern human viruses showed little disease and limited viral replication. The lungs of animals infected with the 1918 HA/NA construct showed bronchial and alveolar epithelial necrosis and a marked inflammatory infiltrate, which suggests that the 1918 HA (and possibly the NA) contain virulence factors for mice. The viral genotypic basis of this pathogenicity is not yet mapped. Whether pathogenicity in mice effectively models pathogenicity in humans is unclear. The potential role of the other 1918 proteins, singularly and in combination, is also unknown. Experiments to map further the genetic basis of virulence of the 1918 virus in various animal models are planned. These experiments may help define the viral component to the unusual pathogenicity of the 1918 virus but cannot address whether specific host factors in 1918 accounted for unique influenza mortality patterns.

## Why Did the 1918 Virus Kill So Many Healthy Young Adults?

**Figure 2:**
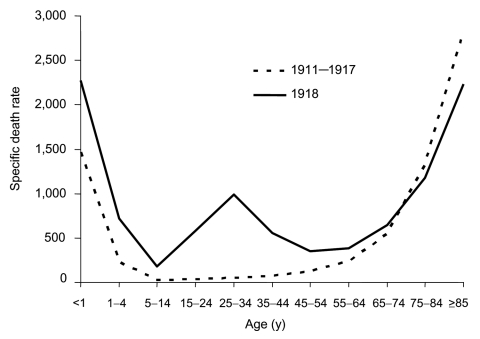
"U-" and "W-" shaped combined influenza and pneumonia mortality, by age at death, per 100,000 persons in each age group, United States, 1911–1918. Influenza- and pneumonia-specific death rates are plotted for the interpandemic years 1911–1917 (dashed line) and for the pandemic year 1918 (solid line) (*33**,**34*).

The curve of influenza deaths by age at death has historically, for at least 150 years, been U-shaped (Figure 2), exhibiting mortality peaks in the very young and the very old, with a comparatively low frequency of deaths at all ages in between. In contrast, age-specific death rates in the 1918 pandemic exhibited a distinct pattern that has not been documented before or since: a "W-shaped" curve, similar to the familiar U-shaped curve but with the addition of a third (middle) distinct peak of deaths in young adults ≈20–40 years of age. Influenza and pneumonia death rates for those 15–34 years of age in 1918–1919, for example, were >20 times higher than in previous years (*35*). Overall, nearly half of the influenza-related deaths in the 1918 pandemic were in young adults 20–40 years of age, a phenomenon unique to that pandemic year. The 1918 pandemic is also unique among influenza pandemics in that absolute risk of influenza death was higher in those <65 years of age than in those >65; persons <65 years of age accounted for >99% of all excess influenza-related deaths in 1918–1919. In comparison, the <65-year age group accounted for 36% of all excess influenza-related deaths in the 1957 H2N2 pandemic and 48% in the 1968 H3N2 pandemic (*33*).

**Figure 3:**
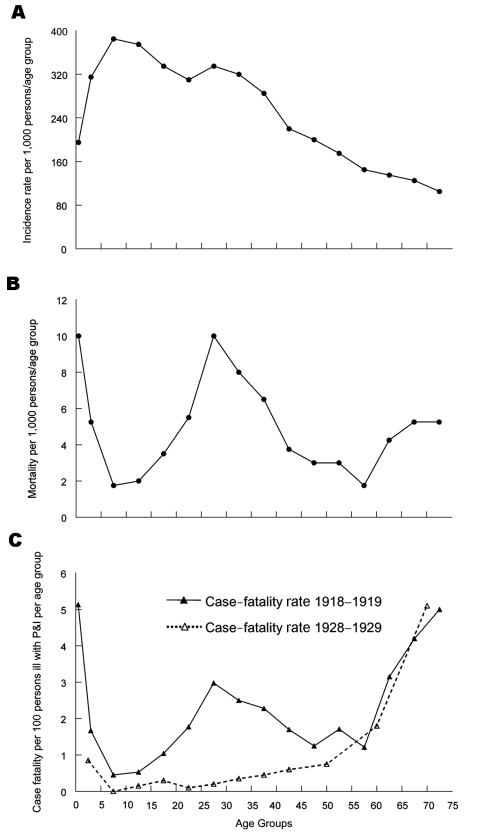
Influenza plus pneumonia (P&I) (combined) age-specific incidence rates per 1,000 persons per age group (panel A), death rates per 1,000 persons, ill and well combined (panel B), and case-fatality rates (panel C, solid line), US Public Health Service house-to-house surveys, 8 states, 1918 (*36*). A more typical curve of age-specific influenza case-fatality (panel C, dotted line) is taken from US Public Health Service surveys during 1928–1929 (*37*).

A sharper perspective emerges when 1918 age-specific influenza morbidity rates (*21*) are used to adjust the W-shaped mortality curve (Figure 3, panels, A, B, and C [35,37]). Persons <35 years of age in 1918 had a disproportionately high influenza incidence (Figure 3, panel A). But even after adjusting age-specific deaths by age-specific clinical attack rates (Figure 3, panel B), a W-shaped curve with a case-fatality peak in young adults remains and is significantly different from U-shaped age-specific case-fatality curves typically seen in other influenza years, e.g., 1928–1929 (Figure 3, panel C). Also, in 1918 those 5 to 14 years of age accounted for a disproportionate number of influenza cases, but had a much lower death rate from influenza and pneumonia than other age groups. To explain this pattern, we must look beyond properties of the virus to host and environmental factors, possibly including immunopathology (e.g., antibody-dependent infection enhancement associated with prior virus exposures [38]) and exposure to risk cofactors such as coinfecting agents, medications, and environmental agents.

One theory that may partially explain these findings is that the 1918 virus had an intrinsically high virulence, tempered only in those patients who had been born before 1889, e.g., because of exposure to a then-circulating virus capable of providing partial immunoprotection against the 1918 virus strain only in persons old enough (>35 years) to have been infected during that prior era (*35*). But this theory would present an additional paradox: an obscure precursor virus that left no detectable trace today would have had to have appeared and disappeared before 1889 and then reappeared more than 3 decades later.

Epidemiologic data on rates of clinical influenza by age, collected between 1900 and 1918, provide good evidence for the emergence of an antigenically novel influenza virus in 1918 (*21*). Jordan showed that from 1900 to 1917, the 5- to 15-year age group accounted for 11% of total influenza cases, while the >65-year age group accounted for 6% of influenza cases. But in 1918, cases in the 5- to 15-year-old group jumped to 25% of influenza cases (compatible with exposure to an antigenically novel virus strain), while the >65 age group only accounted for 0.6% of the influenza cases, findings consistent with previously acquired protective immunity caused by an identical or closely related viral protein to which older persons had once been exposed. Mortality data are in accord. In 1918, persons >75 years had lower influenza and pneumonia case-fatality rates than they had during the prepandemic period of 1911–1917. At the other end of the age spectrum (Figure 2), a high proportion of deaths in infancy and early childhood in 1918 mimics the age pattern, if not the mortality rate, of other influenza pandemics.

## Could a 1918-like Pandemic Appear Again? If So, What Could We Do About It?

In its disease course and pathologic features, the 1918 pandemic was different in degree, but not in kind, from previous and subsequent pandemics. Despite the extraordinary number of global deaths, most influenza cases in 1918 (>95% in most locales in industrialized nations) were mild and essentially indistinguishable from influenza cases today. Furthermore, laboratory experiments with recombinant influenza viruses containing genes from the 1918 virus suggest that the 1918 and 1918-like viruses would be as sensitive as other typical virus strains to the Food and Drug Administration–approved antiinfluenza drugs rimantadine and oseltamivir.

However, some characteristics of the 1918 pandemic appear unique: most notably, death rates were 5–20 times higher than expected. Clinically and pathologically, these high death rates appear to be the result of several factors, including a higher proportion of severe and complicated infections of the respiratory tract, rather than involvement of organ systems outside the normal range of the influenza virus. Also, the deaths were concentrated in an unusually young age group. Finally, in 1918, 3 separate recurrences of influenza followed each other with unusual rapidity, resulting in 3 explosive pandemic waves within a year's time (Figure 1). Each of these unique characteristics may reflect genetic features of the 1918 virus, but understanding them will also require examination of host and environmental factors.

Until we can ascertain which of these factors gave rise to the mortality patterns observed and learn more about the formation of the pandemic, predictions are only educated guesses. We can only conclude that since it happened once, analogous conditions could lead to an equally devastating pandemic.

Like the 1918 virus, H5N1 is an avian virus (*39*), though a distantly related one. The evolutionary path that led to pandemic emergence in 1918 is entirely unknown, but it appears to be different in many respects from the current situation with H5N1. There are no historical data, either in 1918 or in any other pandemic, for establishing that a pandemic "precursor" virus caused a highly pathogenic outbreak in domestic poultry, and no highly pathogenic avian influenza (HPAI) virus, including H5N1 and a number of others, has ever been known to cause a major human epidemic, let alone a pandemic. While data bearing on influenza virus human cell adaptation (e.g., receptor binding) are beginning to be understood at the molecular level, the basis for viral adaptation to efficient human-to-human spread, the chief prerequisite for pandemic emergence, is unknown for any influenza virus. The 1918 virus acquired this trait, but we do not know how, and we currently have no way of knowing whether H5N1 viruses are now in a parallel process of acquiring human-to-human transmissibility. Despite an explosion of data on the 1918 virus during the past decade, we are not much closer to understanding pandemic emergence in 2006 than we were in understanding the risk of H1N1 "swine flu" emergence in 1976.

Even with modern antiviral and antibacterial drugs, vaccines, and prevention knowledge, the return of a pandemic virus equivalent in pathogenicity to the virus of 1918 would likely kill >100 million people worldwide. A pandemic virus with the (alleged) pathogenic potential of some recent H5N1 outbreaks could cause substantially more deaths.

Whether because of viral, host or environmental factors, the 1918 virus causing the first or ‘spring' wave was not associated with the exceptional pathogenicity of the second (fall) and third (winter) waves. Identification of an influenza RNA-positive case from the first wave could point to a genetic basis for virulence by allowing differences in viral sequences to be highlighted. Identification of pre-1918 human influenza RNA samples would help us understand the timing of emergence of the 1918 virus. Surveillance and genomic sequencing of large numbers of animal influenza viruses will help us understand the genetic basis of host adaptation and the extent of the natural reservoir of influenza viruses. Understanding influenza pandemics in general requires understanding the 1918 pandemic in all its historical, epidemiologic, and biologic aspects.
